# EpiFactors: a comprehensive database of human epigenetic factors and complexes

**DOI:** 10.1093/database/bav067

**Published:** 2015-07-07

**Authors:** Yulia A. Medvedeva, Andreas Lennartsson, Rezvan Ehsani, Ivan V. Kulakovskiy, Ilya E. Vorontsov, Pouda Panahandeh, Grigory Khimulya, Takeya Kasukawa, The FANTOM Consortium, Finn Drabløs

**Affiliations:** ^1^Institute of Personal and Predictive Medicine of Cancer, 08916 Badalona, Spain,; ^2^Department of Computational Biology, Vavilov Institute of General Genetics, Russian Academy of Sciences, 119991 Moscow, Russia,; ^3^Department of Biosciences and Nutrition, Karolinska Institutet, 14183 Huddinge, Sweden,; ^4^Department of Cancer Research and Molecular Medicine, Norwegian University of Science and Technology, NO-7489 Trondheim, Norway,; ^5^Engelhardt Institute of Molecular Biology, Russian Academy of Sciences, 119991 Moscow, Russia,; ^6^Division of Genomic Technologies (DGT), RIKEN Center for Life Science Technologies, 1-7-22 Suehiro-Cho, Tsurumi-Ku, Yokohama 230-0045, Kanagawa, Japan

## Abstract

Epigenetics refers to stable and long-term alterations of cellular traits that are not caused by changes in the DNA sequence *per se*. Rather, covalent modifications of DNA and histones affect gene expression and genome stability *via* proteins that recognize and act upon such modifications. Many enzymes that catalyse epigenetic modifications or are critical for enzymatic complexes have been discovered, and this is encouraging investigators to study the role of these proteins in diverse normal and pathological processes. Rapidly growing knowledge in the area has resulted in the need for a resource that compiles, organizes and presents curated information to the researchers in an easily accessible and user-friendly form. Here we present EpiFactors, a manually curated database providing information about epigenetic regulators, their complexes, targets and products. EpiFactors contains information on 815 proteins, including 95 histones and protamines. For 789 of these genes, we include expressions values across several samples, in particular a collection of 458 human primary cell samples (for approximately 200 cell types, in many cases from three individual donors), covering most mammalian cell steady states, 255 different cancer cell lines (representing approximately 150 cancer subtypes) and 134 human postmortem tissues. Expression values were obtained by the FANTOM5 consortium using Cap Analysis of Gene Expression technique. EpiFactors also contains information on 69 protein complexes that are involved in epigenetic regulation. The resource is practical for a wide range of users, including biologists, pharmacologists and clinicians.

**Database URL**: http://epifactors.autosome.ru

## Introduction

Epigenetics has emerged as an extremely fast-growing area of biomedical research. The term ‘epigenetics’ covers DNA and histone modifications, as well as chromatin remodeling. DNA methylation, one of the key epigenetic mechanisms, is involved in differentiation, pluripotency, aging, memory formation and responses to environmental changes (1–5), and it is associated with repression of transcription when present in promoters and expression activation when present in the gene bodies (reviewed, for example, in Refs. (6,7)). At least three independent DNA methyltransferases, DNMT1, DNMT3A and DNMT3B, can establish DNA methylation. The loss of any of these proteins is lethal in mice (8). How DNA methylation affects transcription is still under debate (9–12). Also, the mechanisms of active DNA demethylation are not completely understood. However, several groups of proteins, such as ten-eleven translocation (TET) proteins and DNA glycosylases have been shown to be involved (13). Also, at least two groups of proteins, methyl-binding domain proteins (14) and CxxC proteins (15), can recognize the unmethylated or methylated state of DNA and transmit signals to other regulatory proteins.

Chromatin modification and remodeling are also vital epigenetic mechanisms. In eukaryotes, the core chromatin structural unit—the nucleosome—is composed of eight histones (two copies of H2A, H2B, H3 and H4) (16). The human genome encodes for more than 70 different histone proteins, expressed differently depending upon the cellular and environmental conditions. In human sperm most of the histones are replaced by protamines, which is essential for the higher DNA condensation in sperm cells (17). Histones are subject to a large number of reversible post-translational modifications. Such modifications can modulate chromatin structure, and they can be recognized by specific proteins or protein domains. Such modifications are crucial for epigenetic regulation of transcription, genome stability, DNA damage response, X chromosome inactivation and formation of epigenetic memory (18–21). Various histone marks tend to co-occur in patterns, usually referred to as the histone code (22), which in turn can be ‘read’ by other proteins (23). So far dozens of genes are known to encode proteins that establish or remove histone modifications in the human genome. Chromatin remodeling is carried out by ATP-dependent complexes, which either move, eject or restructure nucleosomes (24). Histone chaperones, critical for nucleosome assembly following DNA replication, DNA repair and gene transcription, also play an important role in epigenetic regulation (25).

The definition of epigenetic factors is not trivial. In addition to the core epigenetic proteins that initiate, modify and act upon epigenetic modifications as described earlier, there are a range of borderline cases, in particular because these core proteins are part of large networks of gene regulation and complex formation (i.e. through protein–protein interactions). Such borderline cases may include transcription factors (TFs) that regulate genes coding for epigenetic factors, TF-like proteins that recruit epigenetic proteins to specific genomic regions, micro-RNAs that act upon mRNAs for epigenetic proteins and long non-coding RNAs that are involved in genome organization and gene regulation. Genes encoding those regulators can in turn be regulated epigenetically or by specific TFs (26, 27), enormously expanding the regulatory network with no clear boundaries between epigenetic and non-epigenetic regulation. Here we mainly focus on the core epigenetic proteins, but some of the borderline cases also have to be included, in particular if they are part of protein complexes that are important for epigenetic processes.

There is an increasing awareness that we have to consider the cell as composed of molecular complexes, each of which performs an independent, discrete biological function (28). In line with this, it should be emphasized that in particular chromatin remodeling is usually not performed by single protein, but by a protein complex, which serves to activate its members or increase their stability. Recent studies of epigenetic complexes have revealed a substantial diversity of proteins that are involved. Most complexes that previously were considered unique appeared to be members of large complex families, as other proteins from the same family could replace core subunits of the complex. There are several families of well-studied epigenetic complexes in eukaryotes, such as SWI/SNF, ISWI, NuRD, INO80 and PcG. To make the picture even more complicated, some proteins can participate in formation of a variety of different complexes.

Over the past decade, tremendous efforts have been directed towards understanding the epigenetic regulatory mechanisms. It is worth noting that deregulation of epigenetic processes is observed in many complex human diseases, including cancer, neurodegenerative diseases and diabetes (29–31). Lately, chromatin-modifying proteins have been considered as promising drug targets (32). Therefore, the information about epigenetic regulators and their complexes is extremely relevant, not only for understanding fundamental biological processes but also for understanding human disorders. This makes a curated and structured source of such information beneficial not only for biologists but also for clinicians. Currently, several publically available databases provide information on the topic. Below we present a brief overview of them.

Databases on DNA methylation, such as MethDB (33, 34), NGSmethDB (35), MethBank (36), MethylomeDB (36), MethHC (37), MethyCancer (38) and The Cancer Genome Atlas (TCGA) (39), primarily provide information about methylation patterns in various normal and pathological conditions, obtained by different experimental techniques. Yet, detailed information about proteins establishing DNA methylation or performing active DNA demethylation, in particular in combination with relevant expression data in various cells, is missing.

Histones and their modifications in humans (mammals) are partially addressed in Human Histone Modification Database (HHMD) (40), Histone Database (41) and HIstome (42). The HHMD focuses on integration of experimental data on genomic distributions of histone modifications. Therefore the number of modifications covered is limited by the number of available antibodies. Moreover, according to the web site, the last update of the database was published 3 years ago, which makes the information somewhat outdated. Histone Database (41) has an evolutionary focus collecting data on histones in a large number of organisms including human. Yet, data on histone-modifying enzymes are not integrated. The recently developed HIstome database (42) compensates for this disadvantage and combines information on the histone proteins and the histone-modifying enzymes. However, other categories of epigenetic regulators are not covered.

Over the last several years, there has been an increased recognition of the role of chromatin remodelers. As a result, databases with a broader scope have started to appear. Those databases incorporate not only information on histones and histone-modifying enzymes but also include other proteins affecting the chromatin structure without direct histone modifications. Among such databases are ChromDB (43), CREMOFAC (44) and CR Cistrome (45). ChromDB (43), which provides information on chromatin-associated proteins for a broad range of organisms, focuses on extremely well-studied plant genes encoding chromatin remodelers. CREMOFAC (44) focuses on ATP-dependent and -independent chromatin-remodeling factors with reduced information on histone-modifying enzymes. CR Cistrome (45) contains information about both chromatin remodelers and histone-modifying enzymes, but with a primary focus on their interactions based on experimental data (ChIP-Seq) in human and mouse. Although these databases represent a significant step toward a comprehensive epigenetic knowledge base, each of them is still missing some important classes of epigenetic regulators.

Some databases go one step further and try to integrate information on various epigenetic regulators. Among them is PEpiD (46), an epigenetic database that combines the three extensively characterized epigenetic mechanisms (DNA methylation, histone modification and microRNA), implicated in prostate cancer of human, mouse and rat. Also EPITRANS (47) and TCGA (39) should be mentioned, as they integrate epigenome and transcriptome data. Furthermore, there are several databases that incorporate all sorts of epigenetic information, such as the NCBI Epigenomics resource (48, 49), NIH Roadmap Epigenomics Program data resource (50) or ENCODE (27). Although such resources provide enormous possibilities for exploring epigenomic data sets, they are mainly focused on the genomic distribution of epigenetic modifications.

Traditionally, the information about protein interactions is reported in a form of one-to-one interactions [protein–protein interactions (PPIs)]. There are several databases providing users with thousands of PPIs, usually with no distinction between stable (long-time, complex forming) and transient (short-time, reversible and context-dependent) interactions. Yet, recently the focus has changed, and the Complex Portal (51), a complex-based database of protein interactions is now part of the IntAct (52) molecular interaction database. Through Complex Portal protein complexes from model organisms are available to search, view and download. But although complexes in the database are annotated with details about their function, the Complex Portal does not focus on epigenetically related complexes, and therefore information about such complexes is difficult to extract.

Despite the availability of existing resources on epigenetic regulation, there is still a need for a comprehensive database that provides a compilation of functional information about epigenetic regulators and their expression in multiple cell types. In addition, since the role and diversity of epigenetic complexes now is recognized, interactions between epigenetic regulators resulting in formation of stable epigenetic complexes have to be included. Most importantly, comprehensive functional annotation is needed to cover all possible epigenetic functions. To this end, we present EpiFactors; a database encompassing detailed and curated information about 815 proteins and 69 complexes involved in epigenetic regulation. To the best of our knowledge, such ample compilation of information on epigenetic factors is not available in any other public resources.

## Database content and development

To create a complete database of the epigenetic regulators, a decision has to be made on which genes and proteins to include. For this purpose we developed a definition of epigenetic factors. Such definitions are always arbitrary to some extent, although we believe that our definition covers the majority of the genes and proteins involved in epigenetic regulation.

### EpiFactors definitions

We defined epigenetic factors as

- Proteins acting as histones, histone variants or protamines;
- Proteins performing post-translational modifications of histones or recognizing such modifications (histone modification ‘writers’, ‘erasers’ or ‘readers’);
- Proteins changing the general structure of chromatin (performing chromatin remodeling), including
  - Proteins that move, eject or restructure nucleosomes (ATP-dependent chromatin remodelers);
  - Proteins that incorporate histone variants into the nucleosomes.
- Proteins assisting histone folding and assembly;
- Proteins acting upon modifications of DNA or RNA in such a way that it affects gene expression, but not through RNA processing;
- Protein cofactors forming complexes with epigenetic factors, where complex formation is important for the activity.

### Epigenetic complexes

As a starting point, we used the UniProt definition of protein complexes as provided in the ‘function’ field for histone-modifying enzymes and chromatin-remodeling proteins. Unfortunately, many of the techniques used to isolate proteins are essentially disruptive to large protein complexes, and even worse, various popular techniques are disruptive to a different extent, which leads to a disagreement between studies with respect to whether some proteins are stable members of a complex or just demonstrating transient PPIs. Although we could not fully overcome this issue, we used information from the most recent papers and expert opinions to exclude transient interactions and to correct the sets of proteins from each complex accordingly. To specify members of the PCG1 complex family, we used the classification provided by Gao *et al.* (53). Some histone-modifying and chromatin-remodeling complexes were updated according to Schuettengruber *et al.* (54). To improve annotation of all repressive complexes, we used a comprehensive review by Laugesen and Helin (55). A complete list of references is linked to the relevant records of the database.

Recent findings suggest that the borders separating protein complexes are becoming vague (56), making definition of the complexes even more complicated. In that line, we tried to group epigenetic complexes, and therefore the complexes are structured into 19 functional complex families.

### Data sources

To create EpiFactors we merged the most recent and complete sets of genes related to epigenetics from several sources, including the Histone Infobase (42) and selected relevant research papers and reviews (57–60). Data from text mining using MeSHOP (MeSH Over-representation Profiles) was also included (W. Wasserman, personal communication). We searched the UniProt database for histones, protamines, their modifying enzymes, DNA methylation enzymes, and chromatin-remodeling proteins in ‘reviewed’ entries of UniProt using the keywords ‘histone’, ‘protamine’, ‘chromatin’ and ‘methylation’, with ‘human’ as species. We manually extracted information about protein complexes from the ‘function’ field of the histone and DNA-modifying enzymes descriptions, and then searched for other proteins in such complexes using the name of the complex as a keyword in the ‘reviewed’ entries of UniProt. We also searched for and investigated paralogs of all identified proteins. However, only proteins where the role as an epigenetic factor was supported by literature were actually included in EpiFactors. We curated the obtained entries to remove non-specific proteins and add missing ones based on the literature. Through this exhaustive database and literature search we identified 95 histones and histone variants (including protamines), 720 DNA/RNA, histone and chromatin-modifying enzymes and their cofactors, and 69 epigenetically relevant complexes. Publications have been used to check the information on every single protein entry, its function, targets and products (if applicable), and complexes that a protein could be involved in. The database provides 598 unique PubMed references to support the annotation of proteins and complexes.

### Functional classes annotation

An important aspect of the database is to provide a useful and comprehensive functional annotation of epigenetic factors. Such annotation is often implemented by using standard gene ontology (GO), which has a rich vocabulary for describing protein function. However, in this case we wanted a smaller set of terms that is more directly targeted toward key aspects of epigenetics. We therefore developed the following annotation scheme.

#### General layout:

1. Function [optional | terms]
  1.1. Modification (alternative | terms)
  1.2. Modification
  1.3. ...

#### Terms that may be used:

1. DNA modification [cofactor]
  1.1. DNA (methylation | demethylation | hydroxymethylation |…)
2. RNA modification [cofactor]
  2.1. RNA (phosphorylation | deamination | degradation |…)
  2.2. mRNA editing
3. Chromatin remodeling [cofactor]
4. Histone chaperone [cofactor]
5. Histone modification [read | write | erase] [cofactor]
  5.1. Histone (methylation | acetylation | phosphorylation | ubiquitination | sumoylation | GlcNAcylation | citrullination |…)
6. Polycomb group (PcG) protein
7. Scaffold protein
8. TF
  8.1. TF (activator | repressor)

We included TFs only if they have been explicitly shown to have epigenetic function, for example as essential members of histone-modifying complexes.

### Target molecules, targets and product annotation

We also annotated entries (proteins and complexes) with information about targets, if such information was available. We provided three types of target/product annotations, covering molecule, target and product. The molecule may be either chromatin, histone, DNA, RNA or mRNA. The target corresponds to the particular nucleotide or amino acid that is affected. For example, C for DNA or H3K4 for histone. Product annotation provides the output of the reaction, as detailed as possible. This could be, for example, H3K4me or H3K4me3.

### Data processing, integration and links to external resources

Custom scripts were used to parse UniProt, HGNC, Entrez, MGI and Pfam databases. In addition to the annotation mentioned earlier, we provide external links to UniProt, HGNC, Entrez, MGI, Pfam, FANTOM5 SSTAR (Abugessaisa *et al*. in preparation), and other public databases. FANTOM5 SSTAR provides a way to explore cell samples, sites of transcriptional initiation and regulators analysed in the FANTOM5 project (61). Links to the corresponding entry (cell type or gene) of FANTOM5 SSTAR are available from both genes (*via* EntrezID) and expression pages. It is worth noting that some epigenetic factors are also TFs and therefore can bind DNA in a sequence-specific manner. If such sequence preferences are known we provide an external link to a corresponding entry of the HOCOMOCO database, which contains curated models of sequence-specific transcriptional factor binding sites (TFBS) (62).

### Information on proteins/genes

The protein page (for each EpiFactor gene or histone) provides a summary of all available information about the protein and expression profiles of the corresponding gene obtained by Cap Analysis of Gene Expression (CAGE), in particular for 458 human primary cell samples, 255 different cancer cell lines and 134 human post-mortem tissues (61). Expression values are sorted and pre-filtered to have expression higher than 10 TPM (CAGE tags per million, relative log expression normalization), but this threshold can be dynamically adjusted. The quantile rank of the gene among all EpiFactors in a given sample is also provided. The information on the gene expression can also be obtained on a per sample basis through the ‘Expression’ tab. It shows all genes expressed in a particular sample. For each gene we also provide quantile ranks of the gene expression in this sample relatively to all other samples.

For the majority of the proteins the respective mouse ortholog from MGI is provided. Where possible we annotated proteins with functional class/function, complex and target molecule/product (see categories above). We extracted such annotation from relevant publications, and the PubMed ID (PMID) is given in the corresponding field.

### Information on complexes

The complex page provides information on important complexes in epigenetics, including proteins involved in complex formation, the molecular and specific targets and products, as well as relevant PMID references supporting the information. It is worth noting that essential and variable members of the complex (according to UniProt annotation) are marked in different colors.

### Implementation, web interface and visualization

**Figure 1.**
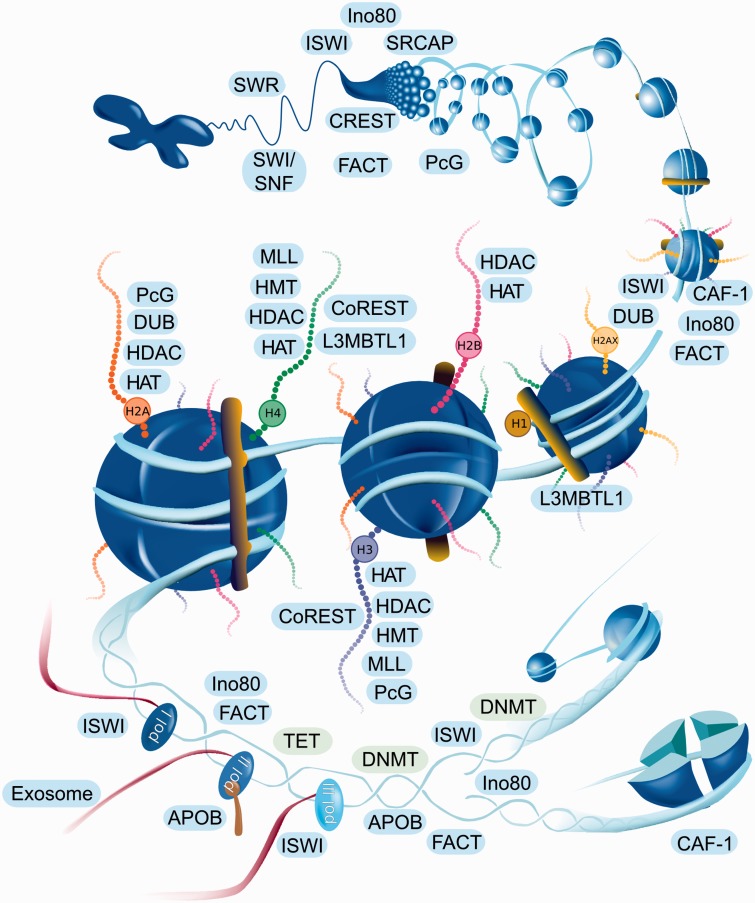
Interactive navigation figure for web page. Names of the histones and complexes are linked to the corresponding entities of the database. Since complexes are represented by a group name, all complexes of the group will be shown when the complex name is clicked on the website navigation figure. Complexes are located in the area corresponding to the shown function. For example CAF-1 (in lower right corner) participates in nucleosome assembly after replication.

EpiFactors is available online *via* a user-friendly web interface implemented as a Ruby-on-Rails front-end with an SQLite back-end. The home page provides a graphical representation of epigenetic processes with all major elements being ‘clickable’ and linked to the corresponding complex groups (Figure 1). The content of the database can also be accessed through four specific tables: ‘Genes’, ‘Complexes’, ‘Histones and protamines’, ‘Expression’, either directly or by using keyword search. Each data table contains a customizable set of columns presenting information on respective entities. A user can also browse individual histones, protamines, epigenetic modifiers and their complexes. We also provide some general information in a ‘Docs’ section of the website, to explain the resource structure and how it can be used.

### Downloads

All tables from the EpiFactors database can be downloaded in csv format. The downloaded file contains all rows and columns that are currently visible, as well as corresponding external links to facilitate downstream analysis. For a particular gene, the expression data can also be downloaded in csv format for all samples where the expression level of the gene is above a selected threshold. Also, expression tables of all the epigenetic factors in all samples can be downloaded from the Download section of the database.

### Summary statistics

The content of the EpiFactors database can be described through some summary statistics. Table 1 shows the number of proteins that have been annotated with each of the main terms for function, and for the type of modification that is targeted (counted across all functions). Please note that some proteins can be annotated with more than one function. The most frequent functions are writing and reading histone modifications, and chromatin remodeling. The most frequent targets are not only, in particular, methylations and acetylations, but also phosphorylations and ubiquitinations.

**Table 1.** Frequency of main terms in annotation

Function	Count	Modification	Count
DNA modification	22	DNA methylation	7
RNA modification	30	DNA demethylation	12
Chromatin remodeling	101	DNA hydroxymethylation	5
Chromatin remodeling cofactor	41	RNA degradation	9
Histone chaperone	26	mRNA editing	10
Histone modification	15	Histone methylation	127
Histone modification cofactor	12	Histone acetylation	139
Histone modification read	90	Histone phosphorylation	55
Histone modification write	158	Histone ubiquitination	61
Histone modification write cofactor	95	Histone sumoylation	2
Histone modification erase	66	Histone citrullination	4
Histone modification erase cofactor	58	TF activator	18
Polycomb group (PcG) protein	29	TF repressor	27
Scaffold protein	12		
TF	53		

**Figure 2.**
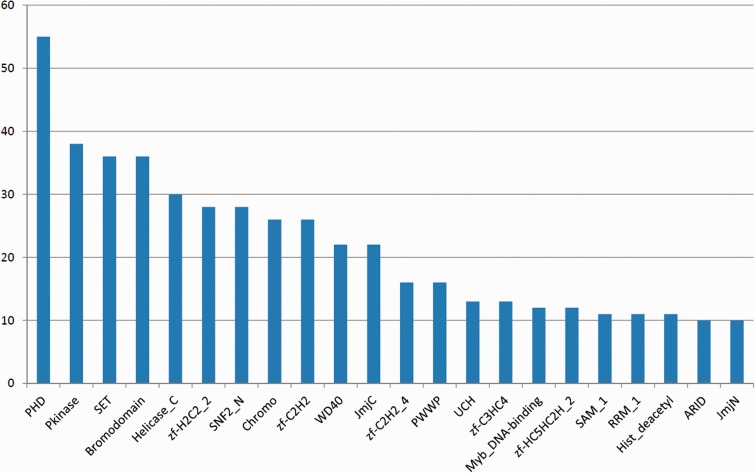
The most frequently occurring Pfam domains in EpiFactors. Multiple occurrences of a domain within the same protein are counted as one occurrence.

Figure 2 shows the most frequent Pfam domains in EpiFactors. Several of these domains are known to be strongly associated with epigenetic processes (see later).

Table 2 shows the most significantly enriched Pfam domains. It shows domains that are enriched in EpiFactors relative to a background of 20 200 reviewed human UniProt entries (left part of table). It also shows domains that are enriched for specific functions within EpiFactors, relative to the whole EpiFactors database (right part of table). These two tests for enrichment are independent, but are shown in a joint table because domains that are significantly enriched within EpiFactors (e.g. the SET domain) also tend to be enriched with respect to a specific function (e.g. writing a histone methylation). All enrichments are significant at *P*<0.05 according to a Fisher’s exact test after Bonferroni correction (not shown), except for Chromo, PHD and PWWP with respect to Function (right part).

**Table 2.** Significantly enriched Pfam domains

Pfam domain	EpiFactors		EpiFactors function; modification	Function	
	In	Out		In	Out
SET	36	2	Histone modification write; Histone methylation	33	3
JmjC	22	0	Histone modification erase; Histone methylation	21	1
JmjN	10	0	Histone modification erase; Histone methylation	9	1
Hist_deacetyl	11	0	Histone modification erase; Histone acetylation	11	0
SNF2_N	28	4	Chromatin remodeling	25	3
Bromodomain	36	2	Histone modification	33	3
zf-HC5HC2H_2	12	0	Histone modification; Histone methylation	12	0
Chromo	26	0	Histone modification read	10	16
PHD	55	11	Histone modification read; Histone methylation	17	38
PWWP	16	6	Histone modification read	7	9

In and Out for EpiFactors (left part of table) represents the number of occurrences that are inside and outside the list of EpiFactors (the ‘outside’ set corresponds to 20 200 reviewed human UniProt entries, minus the EpiFactors entries). In and Out for Function (right part) represents the number of occurrences of the same Pfam domain that are found inside and outside of that particular term in EpiFactors. All enrichments are statistically significant according to a Fisher’s exact test, also after correction for multiple testing, except for the Chromo, PHD and PWWP domains with respect to Function (right part).

For each case in Table 2 we show the number of entries that are inside the set (e.g. in the EpiFactors database) or outside (e.g. in the reviewed UniProt entries not included in EpiFactors). The numbers for inclusion in EpiFactors (left part) show that almost all proteins with the domains listed here are included. We should stress that this information was normally not used when selecting proteins for EpiFactors, which was based almost exclusively on literature data. However, the numbers indicate that we have achieved a good coverage of some important Pfam domains. We see a similar trend for the annotation of Function within EpiFactors (right part), indicating good quality of this annotation. The main exceptions are the Chromo, PHD and PWWP domains, where only subsets of proteins with these domains are associated with specific functions.

The domains listed in Table 2 are mainly as expected. For example, the SET domain is known to be involved in histone methylation (63), the Jmj (Jumonji) domain in histone demethylation (64), the Hist_deacetyl domain in histone deacetylation and the SNF2 domain in chromatin remodeling (65).

## EpiFactors summary

EpiFactors is a web-accessible database that provides broad information about human proteins and complexes involved in epigenetic regulation. It also lists corresponding genes and their expression levels in several samples, in particular 458 human primary cell samples, 255 different cancer cell lines and 134 human post-mortem tissues. Each protein and complex entry has been provided with links to external public resources. We believe that the database will be a valuable tool for researchers working in the rapidly growing field of epigenetics.

## Future developments

The database content is carefully maintained and updated. Repeated literature searches are planned to allow for identification and integration of new entries into the database on a regular basis. A module on association of epigenetic factors with pathological conditions has been planned during expansion phase. We will also consider inclusion of data for other model organisms, to broaden the scope of the database to a larger audience. Any input from groups and individuals with specific areas of epigenetic expertise is welcome.
